# Perflubron Distribution During Transition From Gas to Total Liquid Ventilation

**DOI:** 10.3389/fphys.2018.01723

**Published:** 2018-11-30

**Authors:** Michaël Sage, Symon Stowe, Andy Adler, Claudia Forand-Choinière, Mathieu Nadeau, Claire Berger, Sofia Marouan, Philippe Micheau, Renaud Tissier, Jean-Paul Praud, Étienne Fortin-Pellerin

**Affiliations:** ^1^Departments of Pediatrics and Pharmacology/Physiology, Université de Sherbrooke, Sherbrooke, QC, Canada; ^2^Department of Computer Engineering, Carleton University, Ottawa, ON, Canada; ^3^Department of Mechanical Engineering, Université de Sherbrooke, Sherbrooke, QC, Canada; ^4^Department of Medicine, Université de Poitiers, Poitiers, France; ^5^Department of Pathology, Université de Sherbrooke, Sherbrooke, QC, Canada; ^6^INSERM, Unité 955, Equipe 03, École Nationale Vétérinaire d’Alfort, Université Paris-Est Créteil, Paris, France

**Keywords:** liquid ventilation, perfluorocarbon, electrical impedance tomography, fluoroscopy, newborn lamb

## Abstract

Total liquid ventilation (TLV) using perfluorocarbons has shown promising results for the management of neonatal respiratory distress. However, one important safety consideration for TLV is a better understanding of the early events during the transition to TLV, especially regarding the fate of residual air in the non-dependent-lung regions. Our objective was to assess perflubron distribution during transition to TLV using electrical impedance tomography, complemented by fluoroscopy, in a neonatal lamb model of induced surfactant deficiency. Eight lambs were anesthetized and ventilated in supine position. Surfactant deficit was induced by saline lung lavage. After deflation, lungs were filled with 25 ml/kg perflubron over 18 s, and TLV was initiated. Electrical impedance tomography data was recorded from electrodes placed around the chest, during the first 10 and at 120 min of TLV. Lung perfusion was also assessed using hypertonic saline injection during apnea. In addition, fluoroscopic sequences were recorded during initial lung filling with perfluorocarbons, then at 10 and 60 min of TLV. Twelve lambs were used as controls for histological comparisons. Transition to TLV involved a short period of increased total lung volume (*p* = 0.01) secondary to recruitment of the dependent lung regions. Histological analysis shows that TLV was protective of these same regions when compared to gas-ventilated lambs (*p* = 0.03). The non-dependent lung regions filled with perflubron over at least 10 min, without showing signs of overdistention. Tidal volume distribution was more homogenous in TLV than during the preceding gas ventilation. Perflubron filling was associated with a non-significant increase in the anterior distribution of the blood perfusion signal, from 46 ± 17% to 53 ± 6% (*p* = 0.4). However, combined to the effects on ventilation, TLV had an instantaneous effect on ventilation-perfusion relationship (*p* = 0.03), suggesting better coupling. Conclusion: transition to TLV requires at least 10 min, and involves air evacuation or dissolution in perflubron, dependent lung recruitment and rapid ventilation-perfusion coupling modifications. During that time interval, the total lung volume transiently increases. Considering the potential deleterious effect of high lung volumes, one must manage this transition phase with care and, we suggest using a real-time monitoring system such as electrical impedance tomography.

## Introduction

Total liquid ventilation (TLV), where dense liquid perfluorochemicals (PFC) are used to fill and ventilate the lungs, has promising properties for the management of the extremely immature neonate (Wolfson and Shaffer, 2005). Preterm infants exhibit surfactant deficiency at birth resulting in high surface tension and unstable alveoli with diffuse micro-atelectasis. Conventional gas mechanical ventilation typically used to support these infants generates ventilator-induced lung injury through heterogeneous ventilation (Ramos et al., 2016), volutrauma (Hernandez et al., 1989) and inflammation (Speer, 2006; Ramos et al., 2016). It is also associated with the development of bronchopulmonary dysplasia, a chronic disease with long term morbidity (Gough et al., 2012). TLV use is motivated by evidence of a more homogenous lung recruitment (Hirschl et al., 1994; Tooley et al., 1996) and its potential as an efficient technique to remove pulmonary aspiration debris (Foust et al., 1996; Avoine et al., 2011). Moreover, PFCs have low surface tension, anti-inflammatory properties (Thomassen et al., 1997; Rotta and Steinhorn, 1998; Khalighi et al., 2016) and have been shown to promote lung growth (Nobuhara et al., 1998; Herber-Jonat et al., 2014). PFCs are known for their high solubilities for most gasses (Riess, 2005) enabling them to be used for respiratory support. TLV thus appears as an excellent candidate for bronchopulmonary dysplasia prevention in the most extreme preterm newborns (Wolfson and Shaffer, 2005), and for which animal studies have been positive to date (Wolfson et al., 1992; Degraeuwe et al., 2000; Cox et al., 2003).

The air-liquid interface appears to be completely eliminated during TLV, as gas is entirely evacuated in the ventilator expiratory arm and/or dissolved in PFC (Wolfson and Shaffer, 2005). A post-mortem animal study using CT-scan has indeed provided evidence that the dense PFC is distributed in the entire lung after a period of 1 h of TLV (Hirschl et al., 1994). However, the time interval required to complete this transition is unknown and is potentially affected by the specific filling protocol used. One important safety consideration for TLV is a better understanding of the early events during the transition to TLV, especially regarding the fate of residual air in the non-dependent-lung regions. In our experience, the first few minutes of TLV are the most critical, where most of the instability in hemoglobin oxygen saturation is observed if the lungs are filled too slowly. On the other hand, a rapid insertion of PFC into air-filled lungs could potentially lead to air trapping and overdistention in the non-dependent lung regions, in turn inducing ventilator-induced lung injury. This is particularly important since, in the preterm infant population, even only a few high-volume ventilations at birth have been shown to negatively affect the lungs (Bjorklund et al., 1997). We have also documented a progressive decline in end-expiratory and end-inspiratory pause pressure during the first few minutes of TLV suggesting an increase in lung compliance (Tooley et al., 1996) and/or progressive decrease in total lung volume from air evacuation. Finally, PFC volume must be progressively increased during the first minutes of TLV in order to avoid tracheal collapse during active expirations. After 5 to 10 min, the ventilation is more stable and requires fewer adjustments. These observations have motivated the present study.

To better understand the transition from air to liquid breathing, a bedside, real-time imaging modality was needed. Electrical impedance tomography (EIT) is a non-invasive technique which calculates rapid (4 to 50 images/sec) changing distribution of ventilation using low amplitude electrical current applied to surface electrodes (Frerichs et al., 2017). There is a linear relationship between change in impedance and total lung volume (Adler et al., 1997; Roth et al., 2015). EIT has been successfully used to characterize lung volume during partial liquid ventilation (Wolf et al., 2010), and has also been used to study lung perfusion by analyzing changes in thoracic impedance following the injection of a bolus of hypertonic saline contrast (Borges et al., 2012). EIT is thus a tool with the necessary temporal resolution to describe dynamic processes such as transition to liquid ventilation at bedside. The major limitation of EIT in the context of TLV is that PFC and air both have very low conductivity and they thus cannot be differentiated by EIT. Fortunately, the specific PFC used for this study (perflubron) is radio-opaque such that fluoroscopy can be used to complete the assessment of transition to TLV.

Given the above, our objective was to use EIT and fluoroscopy to ascertain lung recruitment and air evacuation within the first minutes of TLV in a neonatal lamb model of surfactant deficiency. We also aimed to assess how lung perfusion adapts to the distribution of the liquid tidal volume. Our overall goal is to gain insight into the transition phase of TLV in order to avoid ventilator-induced lung injury and assess the need to refine our filling approach.

## Materials and Methods

A total of 20 full-term newborn male lambs were used (3.4 ± 0.2 kg, 3.2 ± 0.5 days of age; 8 in the experimental group, 12 controls), brought to the research facility from a local breeder. The study was approved by the animal research ethics board of the Université de Sherbrooke (protocol # 401-16) and performed in accordance with the Canadian Council on Animal Care guidelines. Experimentations were performed at the animal facility of the Université de Sherbrooke, Canada.

### Anesthesia, Instrumentation and Induction of Surfactant Deficiency

Lambs (*n* = 8) were premedicated with ketamine (10 mg/kg IM) prior to percutaneous cannulation of the left jugular vein. They were intubated with a 4.5 mm cuffed endotracheal tube and placed in supine position. Sixteen EIT electrodes were positioned around the thorax, covering the superior aspect of the posterior lobes. Gas ventilation was initiated (Servo-300; Siemens-Elema, Solna, Sweden) in a pressure-regulated mode, with a positive end-expiratory pressure of 6 cm H_2_O and a peak inspiratory pressure adjusted to generate 7 ml/kg of tidal volume and a backup rate of 40 cycles/min. Hemoglobin saturation was continuously recorded by pulse oximetry (Radical, Masimo, Irvine, CA, United States) with a probe at the base of the tail. Lambs were maintained under general anesthesia throughout the experiment using propofol 120 μg/kg/min and ketamine 1 mg/kg/h, along with a 10% dextrose solution at a rate of 6 ml/kg/h. They received a single IV dose of fentanyl 4 μg/kg before instrumentation. A 3 Fr catheter (PV2013L07, PiCCO catheter; Pulsion Medical System, Munich, Germany) was inserted into the right femoral artery to allow for continuous monitoring of systemic arterial pressure and blood gas measurements. The lambs were paralyzed with rocuronium (0.4 mg/kg iv, 0.2 mg/kg subsequently to maintain paralysis). Lung lavages with 10 ml/kg of warmed saline were performed to induce surfactant deficiency. They were repeated until PaO_2_ reached a value of < 100 Torr (13.33 kPa) under 100% FiO_2_ for at least 20 min.

### Total Liquid Ventilation Protocol

Our TLV filling protocol has been developed over the past few years. The lambs were disconnected from the gas ventilator for 5 s to allow the lung to deflate prior to initiation of TLV under fluoroscopic recording (lateral beam). The lungs were then filled with 25 ml/kg of perfluorooctyl bromide, also called perflubron, at 39°C (Exfluor, Round Rock, TX, United States) over 18 s, using the INOLIVENT-6 liquid ventilator prototype (Nadeau et al., 2016; Sage et al., 2016). In our experience, a slow filling over 20 s is well tolerated by subjects (no oxygen desaturation). We do not preoxygenate the lung to wash nitrogen out, as prolonged period of hyperoxia would not be acceptable for a premature infant, our targeted population, due to risks of oxidative stress. Volume-controlled, pressure-limited and time-cycled TLV was then initiated with a tidal volume of 11–14 ml/kg, a respiratory rate of 6–10 cycles/min, and an inspiratory to expiratory ratio of 1:2. End-expiratory PFC volume was progressively increased over the first five TLV cycles to 30 ml/kg (rate of 1ml/kg/cycle, up from 25 ml/kg just after filling) in order to allow the first cycles to occur at lower lung volume while large volumes of air are being evacuated during expiration. Thereafter, small volumes of PFC (1ml/kg) could be administered by the operator, through the ventilator control unit, as needed to avoid expiratory tracheal collapse (Bagnoli et al., 2013) and compensate for PFC evaporation losses in the ventilator. Respiratory rates are lower than during gas ventilation as PFC has a higher viscosity than air and both inspiratory and expiratory rates need to be decreased to avoid the need for excessive pressure to move the fluid in and out of the subject. Ventilation parameters were aimed at maintaining oxygen saturation > 90% and PaO_2_ > 60 Torr, as well as arterial pH > 7.20 and PaCO_2_ between 50–65 Torr (Ryu et al., 2012). The INOLIVENT-6 ventilator prototype monitors both the inspiratory and expiratory pause pressures as well as the pressure during PFC movement in and out of the subject. EIT signals were recorded during the first 2 h although lambs were subjected to TLV for a total of 5 h. The lambs were then euthanized with one IV injection of 90 mg/kg pentobarbital.

### Control Groups

Two groups of controls were used for histological analysis only. A group of 7 lambs were subjected to the same insult (surfactant deficit) but were maintained under conventional gas ventilation for the entire length of the experiment. Initial ventilation parameters were adjusted to maintain blood gas within the same range as the TLV group, using a permissive hypercapnia approach (Ryu et al., 2012). Another group of 5 newborn lambs (2.8 +/- 0.8 days of age) were euthanized after venous cannulation and lung samples were taken as control for histological analysis as well (i.e., not subjected to mechanical ventilation or lung lavage).

### Electrical Impedance Tomography Monitoring

**FIGURE 1:**
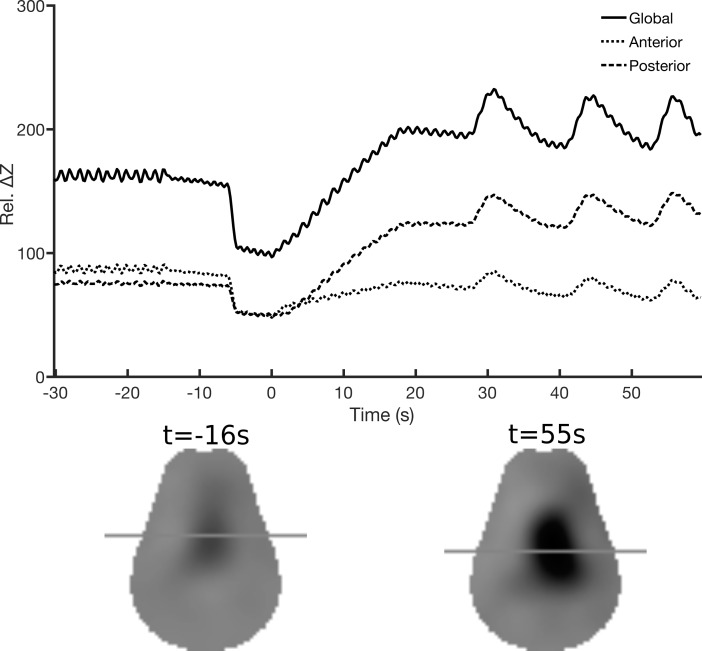
Electrical impedance tomography. Example of the EIT data collected from one lamb during filling with perflubron, which begins at time = 0s. The upper panel shows the average relative impedance change from the reference frame (Rel. ΔZ) for the pixels in the anterior (dotted line) and posterior (dashed line) lung regions during gas ventilation (–30 to –16 s) and total liquid ventilation (from 28 to 55 s). The solid line is the sum of these two lines. The lower panel shows the EIT images reconstructed to show the difference between the end-expiration and end-inspiration (i.e., proxy for tidal volume) during gas ventilation (left) and total liquid ventilation (right). The horizontal lines indicate the position of the vertical center of gravity of the reconstructed image amplitude and illustrate that liquid (vs. gas) ventilation has a larger component in the dependent lung.

EIT uses multiple electrodes positioned around the thorax to reconstruct a 2D image of a transversal slice of the thorax based on the variation of the electrical impedance during ventilation. These images are generated multiple times per second (Figure 1). Measurements were taken with the Sigma tome II (École Polytechnique de Montréal, Montreal, QC, Canada) (Robitaille et al., 2009) device using an applied current at 50 kHz, with an acquisition rate of 4.7 images/sec. Sixteen electrodes (Kendall 7605 Cloth electrodes, Covidien, Dublin, Ireland) where placed in a transversal plane around the thorax of the animals, behind the forelegs. The electrodes were covered with gel to improve contact. Two EIT recordings were made during the experiment: from 5 min before filling up to and including the first 10 min of TLV; and after 120 min of TLV. The EIT belt was removed after this recording.

EIT data were reconstructed using the GREIT algorithm (Adler et al., 2009), set to a noise figure of 0.5, and adapted to the Sigma tome II system and the lamb’s thorax geometry as described below. The Sigma tome system acquires data using an adjacent stimulation and measurement protocol with 416 measurements per frame made after analog filtering of the data. The sensitivity matrix was adapted to account for this filter within the reconstruction algorithm. Reconstruction of a 2D “slice” at the electrode plane was calculated using a segmented lamb CT-based finite element model. Cross-sectional images of the lungs were generated from impedance variations from baseline. The baseline frame selected for the experiments was the one immediately before the subject was disconnected from the gas ventilator, before filling with PFC. The frames were divided into two equal zones (anterior, or non-dependent; and posterior, or dependent regions). The pixels within each of these two regions were summed and averaged over three respiratory cycles in order to measure the contribution of each section (Figure 1).

Absolute changes in impedance values are reported as well as regional ventilation, expressed as a percentage of the global signal change (Frerichs et al., 2006). Measurements were taken at end-expiration. To assess tidal volume distribution, the impedance at end-expiration was subtracted from the end-inspiration value and divided by the total impedance in order to obtain a regional percentage of distribution. In this study, it is the relative change with respect to the “arbitrary” reference impedance level that is analyzed as a proxy for total lung volume (Frerichs et al., 2017). Both the PFC used (perflubron) and air effectively have infinite resistivity with respect to tissue values and cannot be differentiated using EIT (our measurements show it has resistivity greater than 1 MΩ-m with both direct current and alternating current for frequencies below 100 kHz). The saline solution used for lung lavage is immiscible in PFC. In bench impedance measurements, PFC was not affected by the injection of the saline solution and constant agitation of the fluids.

Various techniques have been developed to assess lung perfusion based on EIT. In this study, we used the variation of thoracic impedance with a central venous injection of hypertonic saline during an apnea to describe lung perfusion (Borges et al., 2012). Two-ml injections of hypertonic saline (7%) were administered during an expiratory pause during gas ventilation, and thereafter at 1, 5, 10 and 120 min of TLV to allow regional blood perfusion measurements. The choice of a 2 ml injection was based on a previous pilot study (Adler et al., 2016) that showed adequate impedance change to assess perfusion. The reference frame for this analysis was selected immediately before the injection, during apnea. After the injection, the frame with the widest transversal thoracic distribution of the contrast was used to assess impedance change associated with blood distribution. This method was used to avoid the early right ventricular first pass (Borges et al., 2012) of the contrast agent as much as possible. The distribution of the hypertonic contrast within the anterior and posterior regions of the lungs was then reported as a percentage of the global signal.

### Fluoroscopic Measurements of Perfluorooctyl Bromide (Perflubron) Distribution Into the Lungs

**FIGURE 2:**
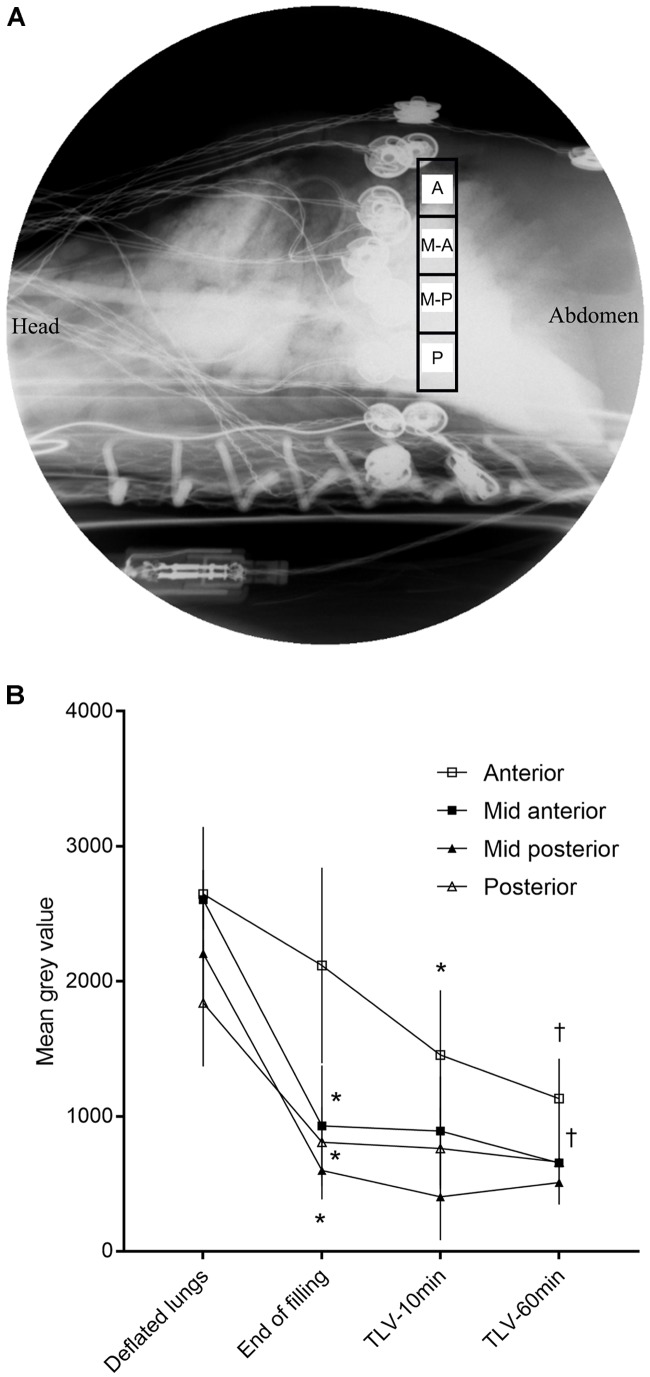
Mean gray values obtained by fluoroscopy. **(A)** Fluoroscopic image of the thorax (lateral beam) showing the 4 regions of interest (A, anterior; M-A, mid-anterior; M-P, mid-posterior; P, posterior) for which a mean gray value was measured (note: image was converted from black to white given that most clinicians are accustomed to associate white with radio-opacity). **(B)** Absolute mean gray value (scale 0 = complete radio-opacity, to 4095 = completely radiolucent) throughout the protocol. A decrease in mean gray value represents an accumulation of perflubron. Values are presented as mean ± SD, *n* = 6. Statistical analysis using Friedman test for repeated measures followed by Wilcoxon signed-rank test. ^∗^First timepoint at which mean gray value differs from the deflated lung value (*p* < 0.05). ^†^Compared to end of filling, the mid-anterior and the anterior region continue to accumulate PFC at 1 h of liquid ventilation (*p* < 0.05).

Lateral beam fluoroscopic recording (model 718095 Philips BV Pulsera, Amsterdam, Netherlands) was performed at 15 frames/s for 30 s throughout the initial lung filling with PFC, then at 10 and 60 min of TLV. The initial settings were selected from a scout image (range 60 to 64 keV, typical current 3.55 mA) for each subject but were subsequently manually fixed to avoid variation throughout the protocols. Due to its radio-opacity, perflubron appears darker than surrounding lung tissue and can be monitored by mean gray values for the pixels within the region of interest (i.e., gray level range 0 to 4095, 0 being complete radio-opacity at the set parameters) using Fiji Image J software^1^. An anteroposterior region of interest, located just caudally to the EIT electrodes, was manually divided into four equal-sized regions for analysis (60 pixels width, 16.8 mm; height dependent on antero-posterior diameter) (Figure 2A). Electrodes used for EIT were usually not protruding in the regions of interest. However, if they were partly within the field, no special treatment was applied to the images. This should not affect the results as each region of interest was only compared to itself throughout the protocol.

### Histological Scoring for Lung Inflammation

Samples from the anterior middle lobe and posterior lower lobe region of the right lung were collected and fixed in formaldehyde 10%. The tissues were then embedded in paraffin from which 5 μm sections were prepared before hematoxylin/eosin staining. A histological score of lung inflammation, previously developed for the newborn lamb, was performed by a blinded pathologist (SM) (Hillman et al., 2010). Four key components (septation thickness, hemorrhage, inflammation cell infiltration, epithelial sloughing) were assessed, each on a scale of 0 to 2 (total worst score of 8).

### Variables and Statistical Analysis

The Friedman non-parametric test for repeated measures followed by the Wilcoxon signed-rank test were used for EIT and fluoroscopy data. Differences between the anterior and posterior regions were analyzed using the Mann-Whitney *U*-test. The Kruskal-Wallis test followed by the Mann-Whitney *U*-test were used for histological scoring assessment. Data are reported as mean ± SD unless specified otherwise. Statistical analyses were performed using SPSS 19 (IBM, Armonk, NY, United States).

## Results

The eight lambs reached entry criteria after a mean of 7 ± 7 lung lavages. Half (50 ± 35%) of the instilled saline was recovered by suction. PaO_2_/FiO_2_ decreased significantly after the insult, as did the other blood gasses parameters (Table 1). During the initial phases of TLV, pressures at end-inspiration and end-expiration decreased rapidly (Table 2).

**Table 1:** Blood gas through the protocol.

	Baseline	Insult	TLV-30 min	TLV-120 min	*p*
PaO_2_	77.9 ± 26.1	74.7 ± 18.7	91.3 ± 52.9	128,9 ± 39,7^∗,†^	0.04
PaO_2_/FiO_2_	274 ± 87	75 ± 19^∗^	-	-	-
PaCO_2_, (mmHg)	42.7 ± 7.2	60.1 ± 7.8^∗^	60.6 ± 6,4^∗^	56.6 ± 3.8^∗^	0.005
pH	7.28 ± 0.12	7.10 ± 0.09^∗^	7.07 ± 0.11^∗^	7.13 ± 0.11^*,#^	<0.001
HCO_3_^-^ (mM)	18.9 ± 4.9	15.3 ± 3.2^∗^	14.5 ± 4.1^∗^	16.0 ± 3.6^*,#^	0.02


*Blood gas evolution through the protocol. Differences were first assessed with a Friedman test for repeated measures followed by a Wilcoxon signed-rank test (^∗^if *p* < 0.05 when compared to baseline value, ^†^if *p* < 0.05 when compared to post insult value, ^#^when compared to TLV-30 min value).*

**Table 2:** Ventilation parameters through the protocol.

	Baseline	Insult	*p*	TLV-1 min	TLV-30 min	TLV-120 min	*p*
Vt (ml/kg)	6.6 ± 1.3	6.2 ± 2.4	0.5	12.4 ± 0.2	13.6 ± 1.0^∗^	14.6 ± 1.3^∗,†^	0.03
RR (/min)	49 ± 13	51 ± 6.1	0.3	6.7 ± 0.6	9.3 ± 0.7^∗^	8.8 ± 0.5^∗^	0.002
PIP/EIPP (cmH_2_O)	14.5 ± 1.7	18.3 ± 2.3	0.04	17.0 ± 2.6	14.7 ± 2.3^∗^	11.8 ± 3.2^∗,†^	0.002
PEEP/EEPP (cmH_2_O)	5.1 ± 0.6	6.6 ± 2.2	0.02	12.2 ± 2.0	4.2 ± 3.0^∗^	0.7 ± 4.1^∗,†^	0.001


*Gas ventilation parameters, at baseline and after the insult, are reported for 8 animals and compared using a Wilcoxon signed-rank test. Liquid tidal volume (Vt), respiratory rate (RR), end-expiratory pause pressure (EEPP) could only be recorded in 7 subjects and end-inspiratory pause pressure (EIPP) in 6 during total liquid ventilation (TLV) due to technical problems. Differences for total liquid ventilation parameters were assessed with a Friedman test for repeated measures followed by a Wilcoxon signed-rank test (^∗^if *p* < 0.05 when compared to TVL-1 min value, ^†^if *p* < 0.05 when compared to TVL-30 min value). Data are presented as mean ± SD. PIP, peak inspiratory pressure during gas ventilation. PEEP, positive end-expiratory pressure during gas ventilation.*

### Monitoring of Lung Volume With Electrical Impedance Tomography

**FIGURE 3:**
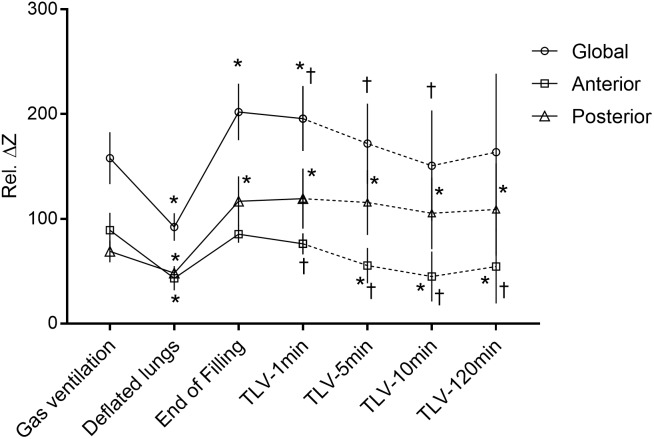
End-expiratory lung volumes assessed by electrical impedance tomography. Total and regional lung volumes (air + perflubron) assessed by electrical impedance tomography during the transition from gas to total liquid ventilation. The change in impedance relative to the reference value is indicated by the y-axis (see method section). A higher value on the Y-axis should be interpreted as a higher lung volume. ^∗^Different than during gas ventilation (*p* < 0.05). ^†^Different from end-filling value, for timepoints after filling (*p* < 0.05). There was no significant difference between the 10-min values and the 120-min values for the three curves. Values are presented as mean ± SD. Statistical analysis using Friedman test for repeated measures followed by Wilcoxon signed-rank test (*n* = 8). TLV: total liquid ventilation.

EIT signal was acquired for all 8 lambs in the TLV experimental group. Total lung volume decreased when the subjects were disconnected from the gas ventilator, just before filling the lungs with PFC (*p* = 0.01) (Figure 3). With filling (25 ml/kg), global impedance reached higher values than during gas ventilation (+29 ± 13%, *p* = 0.01). During the first few liquid breathing cycles, visible large gas bubbles were expelled through the endotracheal tube. Despite an increase in end-expiratory PFC volume left in the lungs during the first minute of TLV, from 25 ml/kg to 30 ml/kg, end-expiratory global impedance decreased slightly during this same time interval (*p* = 0.02), suggesting that more air is evacuated than PFC entering the lungs. Global impedance continued to decrease between the first and the tenth minute of TLV (-24 ± 17%, *p* = 0.02), stabilizing thereafter. The impedance signal was the same at 10 min and 120 min TLV, with a value similar to that measured during gas ventilation (*p* = 0.4).

The increase in total lung volume during the initial filling relied exclusively on the posterior, or dependent, lung regions. In the anterior regions, no significant change in volume was observed between gas ventilation and the end of lung filling with PFC. Moreover, the anterior impedance values measured 1 min after TLV initiation were lower than during gas ventilation, although the difference did not reach statistical significance (*p* = 0.05). Conversely, lung volume in the posterior region increased by 70 ± 28% (*p* = 0.01) from gas ventilation to the end of lung filling with PFC and remained stable thereafter (Figure 3). The proportion of global signal attributed to the anterior regions of the lung decreased progressively from 56 ± 4% during gas ventilation to 33 ± 8% at 5 min of TLV (*p* = 0.01), remaining stable thereafter (31 ± 11% at 120 min, *p* = 0.7).

### Fluoroscopic Assessment of PFC Distribution at End-Expiration

Fluoroscopic sequences were obtained for 6 of 8 lambs due to the availability of the equipment. The most anterior lung region filled slowly while the other regions (mid-anterior, mid-posterior and posterior) received most of the initial 25 ml/kg PFC filling (Figures 2A,B). The two anterior (anterior and mid-anterior) regions filled significantly between the end of filling and 60 min of TLV. It is however, not clear if significant filling occurred after the first 10 min (1524 ± 467 vs. 1278 ± 372 mean gray value at 10 min vs. 60 min for the most anterior region, *p* = 0.1).

### Distribution of Tidal Volume and Lung Perfusion

**FIGURE 4:**
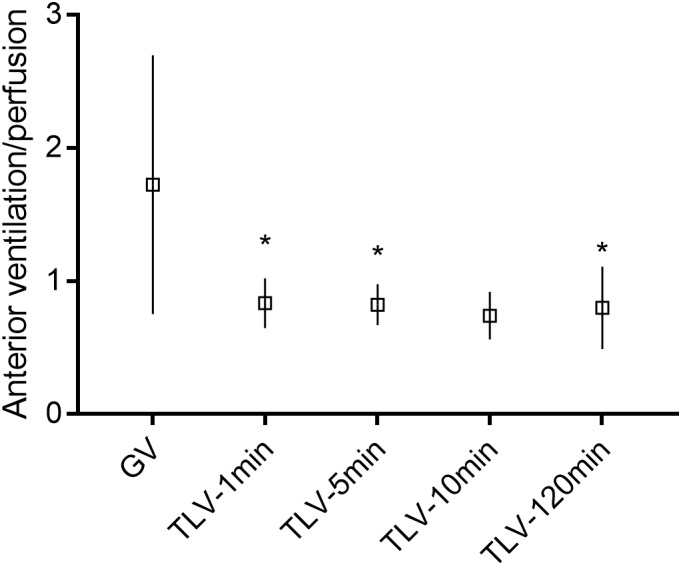
Anterior lung ventilation-perfusion ratio. In this figure, the electrical impedance tomography proxy for the tidal volume is divided by the proxy for the perfusion (for anterior lung regions, the ratio is shown on the *Y*-axis). The proxy used to assess the anterior tidal volume is the impedance change during inspiration in the anterior regions divided by the global lung impedance change during inspiration (i.e., approximating the percentage of tidal volume going to the anterior lung regions). The proxy for perfusion was obtained by injecting a bolus of hypertonic saline during an apnea (i.e., impedance variation in the anterior lung regions divided by the global change in impedance). GV, gas ventilation; TLV, total liquid ventilation. ^∗^Different from initial value during gas ventilation, analyzed by Friedman test for repeated measures (GV, 1 min, 5 min, and 120 min) followed by Wilcoxon signed-rank test, *n* = 7, *p* < 0.05. Recordings from one animal were of poor quality during the saline injection and were not analyzed. The 10-min value was excluded from the analysis as only four animals were recorded. The posterior regions have the exact opposite pattern and are thus not shown in the figure.

During gas ventilation, most of the tidal volume was directed to the anterior half of the lungs but this changed rapidly with TLV (66 ± 8% to 46 ± 12%, during gas ventilation and at 1 min after TLV initiation, respectively, *p* = 0.02). The distribution of tidal volume was stable thereafter (47 ± 19% at 120 min of TLV, *p* = 0.2 when compared to the 1-min value). Perfusion exhibited a different pattern. For this analysis, data from only 7 animals could be used because recordings from one animal were of poor quality during the saline injection. During gas ventilation, only 46 ± 17% of the EIT signal change occurred in the anterior region with hypertonic saline injection. PFC filling was associated with a non-significant increase in the anterior distribution of the perfusion signal to 53 ± 6% (*p* = 0.4 vs. gas ventilation), which remained stable thereafter (54 ± 4% at 2 h of TLV). However, considering the reciprocal changes in tidal volume distribution, the ventilation-to-perfusion relationship changed with TLV (*p* = 0.004) (Figure 4). Although this is not an exact measurement of the ventilation-perfusion ratio, it provides evidence that this ratio is affected by total liquid ventilation as soon as it is initiated. Considering the known ventilation-perfusion anomalies in this pathologic model, these changes may positively affect ventilation-perfusion coupling (Morris et al., 2000; Riedel et al., 2009).

### Histological Scoring of Lung Damage

**FIGURE 5:**
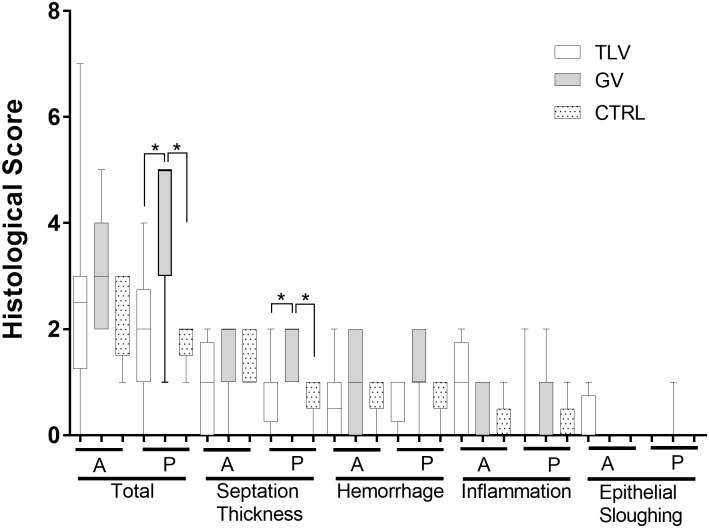
Pathological assessment of lung inflammation. Three groups were used to assess the impact of total liquid ventilation in this pathological neonatal model, using the Hillman histological score. TLV (total liquid ventilation, *n* = 8) represents the group of lambs studied with electrical impedance tomography and fluoroscopy while CTRL represents a control group where lambs were not mechanically ventilated or subjected to the insult (*n* = 5). The lambs from the gas ventilation group (GV, *n* = 7) were subjected to the same insult as the TLV group but were maintained under conventional gas ventilation for the entire duration of the experiment. A, anterior regions of the lung; P, posterior lung regions. ^∗^Significantly different (*p* < 0.05). One animal from the TLV group was only subjected to TLV for 3 h due to a technical problem with the liquid ventilator prototype. The exclusion of this subject does not affect the statistical results presented. A Kruskal Wallis test followed by Mann-Whitney *U*-test was used.

No significant changes in the histological score were found between TLV lambs and control lambs (Figure 5) despite the insult. Conversely, the score was higher (i.e., more damage) in the group subjected to the insult but maintained on conventional gaseous ventilation. This last group received the same number of lung lavages before they reached the entry criteria (6.9 ± 5, *p* = 0.7) with a similar deterioration of blood gasses (pH 7.26 ± 0.05 vs. 7.13 ± 0.09, *p* = 0.02; paCO_2_ 41 ± 9 vs. 57 ± 10 Torr, *p* = 0.03; HCO_3_^-^ 18 ± 4 vs. 16 ± 4 mmol/l, *p* = 0.08, for baseline and post-insult values, respectively) and PaO_2_/FiO_2_ (284 ± 124 vs. 70 ± 13, *p* = 0.02). The blood gas values of both groups were similar during the protocol except for a lower PCO_2_ in the GV group at 5 h (59 ± 7 vs. 45 ± 6 torr, *p* = 0.001). These animals were maintained under invasive conventional mechanical ventilation and peak inspiratory pressures were adjusted to maintain adequate tidal volumes after the insult (peak inspiratory pressure 16 ± 3 vs. 21 ± 3 cmH_2_O, *p* = 0.02; tidal volume 6.5 ± 1.3 vs. 5.6 ± 1.2, *p* = 0.06). TLV was mostly beneficial to the dependent lung regions when compared to subjects supported with gas ventilation only.

## Discussion

The current project was aimed at gaining insight into the transition phase of TLV in order to avoid ventilator-induced lung injury. We had a specific concern regarding potential air trapping in the non-dependent lung regions during this transition. In our neonatal lamb model, filling is a dynamic process involving a short period of increased total lung volume secondary to the rapid recruitment of the dependent lung regions. Pathological examination showed that TLV was protective for these same regions. The non-dependent lung regions were progressively filled with PFC but remained under the volume recorded during gas ventilation, suggesting regional overdistention is not occurring. The distribution of the tidal liquid volume, from the start of TLV and thereafter, was more homogenous than during gas ventilation. TLV had an immediate, likely beneficial effect on ventilation/perfusion relationship. Overall, our rapid filling approach was very well tolerated by the animals and results from EIT analysis are reassuring.

As expected based on the existing literature on partial liquid ventilation (Quintel et al., 1998; Miller et al., 2001), PFC initially filled the dependent lung regions. Both mathematical models (Tarczy-Hornoch et al., 2000) and post-mortem assessments had suggested a homogenous recruitment of the lung by PFC (Wolfson et al., 1988; Hirschl et al., 1994) at some point after transition. What was unclear was the sequence of events that would lead to stable TLV and elimination of the air-to-liquid interface. Dealing with residual lung air during transition to TLV has been a concern since the early days of this technology. In 1974, Shaffer suggested the use of positioning, thoracic manipulation and nitrogen removal through hyperventilation with 100% oxygen to deal with this issue (Shaffer and Moskowitz, 1974), an approach that would be unacceptable for the preterm infant due to concerns related to oxidative stress. There is in fact very limited evidence suggesting the superiority of a specific filling protocol. In the present study, we show that the anterior regions of the lungs were progressively recruited by PFC over the first 10 min of TLV. Anterior regions had a similar volume to that during gas ventilation just after filling but quickly decreased to lower values as early as 1 min after the initiation of TLV cycling. The gain in total lung volume is thus entirely attributed to recruitment of the dependent lung regions in our full-term ovine model of respiratory distress, in agreement with the literature (Tooley et al., 1996; Gauger et al., 1998). Pathological examination did not show major lung damage in any group, as is expected with a model of lung lavage with saline used in order to induce surfactant deficiency (Ballard-Croft et al., 2012). It however, showed some protective effect of TLV for the dependent lung regions. We speculate that alveolar recruitment and avoidance of shear stress induced by repeated reopening injury are protective to these regions, known for their tendency to atelectasis (Bhatia et al., 2017). Regional lung volume assessed by EIT as well as the amount of PFC assessed by fluoroscopy in the non-dependent lung regions appeared to be relatively stable between 10 and 120 min of TLV, suggesting that most of the transition was completed by 10 min of TLV. Continuing slow replacement of air by PFC in the anterior regions after 10 min is however, not excluded as this study was not powered to detect very small differences in mean gray values during fluoroscopy.

The present study also demonstrates for the first time that tidal volume, and not only lung recruitment measured at end-expiration, was more homogeneously distributed during TLV as compared to gas ventilation. The redistribution of blood flow to the non-dependent regions, described in previous partial liquid ventilation studies (Doctor et al., 1998; Morris et al., 2000; Harris et al., 2002), was in this instance modest and did not reach significance. However, given the opposite effects on tidal volume distribution and blood flow, TLV initiation immediately affected the ventilation-perfusion coupling and was stable thereafter. Studies on preterm infants have shown that a larger proportion of gas ventilation is distributed to the non-dependent lung regions (Riedel et al., 2009) whereas pulmonary blood flow distribution in mechanically ventilated subjects is predominantly directed in the dependent regions (Morris et al., 2000). Infants with respiratory distress syndrome thus have a significant ventilation-perfusion mismatch (Hand et al., 1990; Billman et al., 1994). Our findings suggest TLV could positively affect such ventilation-perfusion mismatch in this population. However, the latter will need to be evaluated properly as our assessment is based on proxies, and thus we cannot conclude at this point that the optimal ventilation-perfusion ratio obtained herein with EIT is indeed the ultimate optimal value.

### Limitations

The choice of a term neonatal lamb model of induced surfactant deficiency, as a preliminary step before moving to the preterm lamb model, was made in part because of its robustness, availability and lower cost. In addition, lung lavage using saline is a recognized model of induced surfactant deficiency (Merz et al., 2001) and mimics neonatal respiratory distress syndrome by inducing a heterogeneous and low compliant lung (Mokra and Calkovska, 2017). One could argue that since we only recovered approximately half of the saline injected into the lung, the residual saline could have affected our results. We believe the very significant increase in impedance observed in the dependent lung regions during PFC filling provides evidence that residual saline did not prevent lung recruitment in these regions. Hypertonic saline injection could have affected electrical properties of the tissue, at least during a few heart beats, decreasing the global impedance (i.e., and thus suggesting lower volumes). The 5 and 10-min data points would have been the most sensitive to such bias. To limit the effects of this phenomenon, the ventilation EIT signal was systematically recorded prior to the hypertonic injection. We believe our recording at 2 h, showing a similar lung volume as that during gas ventilation, was probably not significantly affected by the saline injection at 10 min. We additionally chose to present some of the EIT data as a percentage of the global signal in order to negate any potential effect of hypertonic injection on regional impedance recordings. The PFCs are inert and generally considered safe for human use (Riess, 2005). However, there is insufficient data on long term effects of TLV with PFC in the neonatal population and further studies are required before a large-scale human study can be initiated.

## Conclusion

Filling the lungs with PFC involves rapid (i.e., dependent lung region recruitment, ventilation-perfusion changes) and slow phenomena (i.e., non-dependent lung PFC filling and air evacuation) occurring over at least 10 min. The findings herein provide reassuring evidence that no damageable regional overdistention resulted from our filling protocol.

## Author Contributions

ÉF-P, PM, J-PP, RT, and AA provided the original idea. MS, CF-C, SS, CB, MN, and ÉF-P performed the experimentations. MS, CB, SS, AA, RT, ÉF-P, MN, CF-C, SM, PM, and J-PP collected, analyzed and interpreted the data. MS, SS, CB, AA, CF-C, MN, SM, PM, RT, J-PP, and ÉF-P prepared the manuscript.

## Conflict of Interest Statement

PM, MN, and J-PP are co-inventors of patents related to the ventilator prototype used for this study (Apparatus for conducting total liquid ventilation with control of residual volume and ventilation cycle profile, US 7,726,311 B2, EP 1 424 090 B1, CA 2,451,261 AND Indirect measurement in a total liquid ventilation system, PCT/CA2014/205548 US 2016/0271348 A1). RT is inventor of a patent related to the use of liquid ventilation (Method for treatment of a body of a mammal in cardiac arrest, US 9,439,804, B2). The remaining authors declare that the research was conducted in the absence of any commercial or financial relationships that could be construed as a potential conflict of interest.

## Footnotes

1 https://imagej.nih.gov/ij
